# Dexamethasone intravitreal implant in previously treated patients with diabetic macular edema: subgroup analysis of the MEAD study

**DOI:** 10.1186/s12886-015-0148-2

**Published:** 2015-10-30

**Authors:** Albert J. Augustin, Baruch D. Kuppermann, Paolo Lanzetta, Anat Loewenstein, Xiao-Yan Li, Harry Cui, Yehia Hashad, Scott M. Whitcup, for the Ozurdex MEAD Study Group

**Affiliations:** Department of Ophthalmology, Staedtisches Klinikum Karlsruhe, Moltkestrasse 90, 76133 Karlsruhe, Germany; Gavin Herbert Eye Institute, University of California at Irvine, Irvine, CA USA; Department of Medical and Biological Sciences – Ophthalmology, University of Udine, Udine, Italy; Tel Aviv Medical Center, Tel Aviv University, Tel Aviv, Israel; Allergan plc, Irvine, CA USA; Avanir Pharmaceuticals, Inc, Aliso Viejo, CA USA; Jules Stein Eye Institute, David Geffen School of Medicine at UCLA, Los Angeles, CA USA

**Keywords:** Corticosteroid, Dexamethasone, Diabetic retinopathy, Drug delivery, Implant, Macular edema

## Abstract

**Background:**

Dexamethasone intravitreal implant 0.7 mg (DEX 0.7) was approved for treatment of diabetic macular edema (DME) after demonstration of its efficacy and safety in the MEAD registration trials. We performed subgroup analysis of MEAD study results to evaluate the efficacy and safety of DEX 0.7 treatment in patients with previously treated DME.

**Methods:**

Three-year, randomized, sham-controlled phase 3 study in patients with DME, best-corrected visual acuity (BCVA) of 34–68 Early Treatment Diabetic Retinopathy Study letters (20/200–20/50 Snellen equivalent), and central retinal thickness (CRT) ≥300 μm measured by time-domain optical coherence tomography. Patients were randomized to 1 of 2 doses of DEX (0.7 mg or 0.35 mg), or to sham procedure, with retreatment no more than every 6 months. The primary endpoint was ≥15-letter gain in BCVA at study end. Average change in BCVA and CRT from baseline during the study (area-under-the-curve approach) and adverse events were also evaluated. The present subgroup analysis evaluated outcomes in patients randomized to DEX 0.7 (marketed dose) or sham based on prior treatment for DME at study entry.

**Results:**

Baseline characteristics of previously treated DEX 0.7 (*n* = 247) and sham (*n* = 261) patients were similar. In the previously treated subgroup, mean number of treatments over 3 years was 4.1 for DEX 0.7 and 3.2 for sham, 21.5 % of DEX 0.7 patients versus 11.1 % of sham had ≥15-letter BCVA gain from baseline at study end (*P* = 0.002), mean average BCVA change from baseline was +3.2 letters with DEX 0.7 versus +1.5 letters with sham (*P* = 0.024), and mean average CRT change from baseline was −126.1 μm with DEX 0.7 versus −39.0 μm with sham (*P* < 0.001). Cataract-related adverse events were reported in 70.3 % of baseline phakic patients in the previously treated DEX 0.7 subgroup; vision gains were restored following cataract surgery.

**Conclusions:**

DEX 0.7 significantly improved visual and anatomic outcomes in patients with DME previously treated with laser, intravitreal anti-vascular endothelial growth factor, intravitreal triamcinolone acetonide, or a combination of these therapies. The safety profile of DEX 0.7 in previously treated patients was similar to its safety profile in the total study population.

**Trial registration:**

ClinicalTrials.gov NCT00168337 and NCT00168389, registered 12 September 2005

## Background

Diabetic macular edema (DME), a common cause of vision loss, is estimated to affect 21 million individuals worldwide [1]. The pathogenesis of DME is multifactorial and not completely understood, but inflammation has a key role [2, 3]. Expression of inflammatory mediators including vascular endothelial growth factor (VEGF), intercellular adhesion molecule-1, interleukin-6, and monocyte chemotactic protein-1 [4], retinal leukostasis [5], and changes in vascular endothelial cell tight junction proteins [6] are involved in the breakdown of the blood-retinal barrier that leads to DME.

Treatment options for fovea-involved DME with vision loss include laser, intravitreal anti-VEGF, and intravitreal corticosteroids. Focal/grid laser photocoagulation of leaking microaneurysms and areas of diffuse capillary bed leakage reduces the risk of moderate vision loss in DME [7], but it is often ineffective in restoring lost vision [8]. Anti-VEGF treatment with ranibizumab is more effective than laser in improving vision in patients with DME [9, 10], and anti-VEGF injections have become preferred first-line treatment for many patients [11]. The dosing frequency of anti-VEGF may be a treatment burden, however, as visual gains are most pronounced when monthly dosing is used (for example, in the phase 3 RISE/RIDE trials) [12]. Furthermore, DME in some patients may be nonresponsive or poorly responsive to anti-VEGF treatment. In the RISE/RIDE trials, approximately 40 % of patients failed to achieve BCVA of 20/40 or better and over 50 % of patients failed to achieve a ≥15-letter gain in BCVA after 2 years of monthly ranibizumab 0.5 mg injections [12]. Additional treatment options/strategies are needed for patients with DME who fail to achieve significant improvement in visual acuity with laser and/or anti-VEGF therapy.

Corticosteroids are a rational treatment for DME because they block the expression of VEGF and other inflammatory mediators of DME [13], inhibit leukostasis [14], improve the barrier function of endothelial cell tight junctions [15], and decrease vascular leakage [14]. Intravitreal injections of triamcinolone acetonide, used off label to treat DME, improve vision in some patients [16]. The most frequent side effects of triamcinolone acetonide treatment are increases in intraocular pressure (IOP) and cataract [16]. In the DRCR.net Protocol I study, triamcinolone acetonide and ranibizumab were similarly effective when combined with laser treatment in pseudophakic eyes [17].

Dexamethasone is a corticosteroid with more potent anti-inflammatory activity than triamcinolone acetonide, but the half-life of dexamethasone in the vitreous is short (<4 h) [18]. A sustained-release dexamethasone implant was developed to reduce the need for frequent intravitreal injections. Dexamethasone intravitreal implant (DEX implant, Ozurdex; Allergan plc, Irvine, CA) is a biodegradable implant that provides sustained, localized release of dexamethasone to the posterior segment. Dexamethasone is released over a period of up to 6 months as the copolymer matrix of the implant degrades into lactic acid and glycolic acid, which are subsequently metabolized to carbon dioxide and water [19, 20]. In 2 randomized, multicenter, sham-controlled phase III clinical trials evaluating DEX implant in patients with DME (the MEAD study), an average of 4 to 5 injections of DEX implant 0.7 mg or 0.35 mg over 3 years provided clinically significant visual and anatomic improvements compared with sham procedure [21]. The safety profile of the implant was favorable. Steroid-induced cataract and increases in IOP occurred as expected, but the IOP increases were usually controlled with topical medication, and only 1 patient in each DEX implant group required glaucoma incisional surgery to control a steroid-induced increase in IOP [21]. These results supported the recent US Food and Drug Administration approval of DEX implant 0.7 mg for treatment of DME in adult patients.

The present analysis was undertaken to evaluate outcomes of DEX implant 0.7 mg treatment in the subset of patients within the MEAD study who were enrolled with DME that had been previously treated with laser and/or medical therapy. Subgroup analysis evaluated efficacy and safety results in subgroups defined by prior treatment of DME in the study eye at baseline.

## Methods

The subgroup analysis used pooled data from 2 randomized, multicenter, masked, sham-controlled, 3-year, phase 3 clinical trials with identical protocols that evaluated the safety and efficacy of DEX implant for treatment of DME. The trials (registered as NCT00168337 and NCT00168389 at ClinicalTrials.gov) were conducted at 131 sites in 22 countries from February 2005 to June 2012. The trials were carried out in accordance with the tenets of the Declaration of Helsinki and were compliant with the Health Insurance Portability and Accountability Act of 1996. An institutional review board or independent ethics committee (105 in total) approved the study protocol at each site, and all patients provided written informed consent. The study protocol was described in detail previously [21] and is summarized here.

### Study participants

Adult patients with diabetes mellitus and vision loss secondary to fovea-involved macular edema associated with diabetic retinopathy were enrolled. Key eligibility criteria for study eyes included BCVA between 34 and 68 letters (20/200 and 20/50 Snellen equivalent) measured with the ETDRS method, central retinal thickness (CRT) in the 1 mm central macular subfield ≥300 μm measured by time-domain optical coherence tomography (OCT) on the OCT2 or OCT3 (Stratus OCT, Carl Zeiss Meditec Inc., Dublin, CA) machine, and either documented history of prior treatment of DME with laser, anti-VEGF, or steroid, or the patient had refused laser, or the investigator believed that the patient would not benefit from laser. Patients with uncontrolled diabetes (glycosylated hemoglobin [HbA1c] >10 %), a history of a marked steroid-induced increase in IOP in either eye, or current or planned use of systemic steroids were excluded. Key exclusion criteria for study eyes included intravitreal anti-VEGF treatment within 3 months of study entry, history of intravitreal steroid other than triamcinolone acetonide, intravitreal triamcinolone acetonide or periocular depot of steroid within 6 months of study entry, intraocular laser or incisional surgery within 90 days of study entry, glaucoma, ocular hypertension (untreated IOP >23 mm Hg, IOP >21 mm Hg treated with 1 antiglaucoma medication, or use of ≥2 antiglaucoma medications), aphakia or an anterior chamber intraocular lens, history of pars plana vitrectomy, and active iris or retinal neovascularization. For patients with both eyes eligible, the eye with the shorter duration of macular edema was selected as the study eye.

### Intervention and visit schedule

At baseline, 1048 patients were randomized in a 1:1:1 ratio to treatment with DEX implant 0.7 mg, DEX implant 0.35 mg, or sham procedure. A single-use applicator system was used to place DEX implant into the vitreous of the study eye through the pars plana [22]. In the sham procedure, a needleless applicator was pressed against the conjunctiva of the study eye. Patients were seen at outcomes assessment visits every 1.5 months during the first year of the study and every 3 months during the second and third year, as well as at safety visits 1, 7, and 21 days after each study treatment or retreatment. The final visit was at month 36 (or month 39 for patients treated at month 36 after a protocol amendment allowing treatment at month 36). Eligibility for retreatment was evaluated every 3 months starting at month 6. Patients were eligible for retreatment if it had been at least 6 months since the previous study treatment, there was evidence of residual edema on OCT (e.g., CRT >225 μm), and in the judgment of the investigator, retreatment would not put the patient at significant risk. The retreatment interval of ≥6 months was selected based on data from a phase 2 study [19] showing that the effects of DEX implant lasted up to 6 months after the intravitreal injection.

No concomitant treatments for macular edema in the study eye were allowed during the study. Patients were required to be withdrawn from the study prior to receiving any escape therapy (medical, laser, or surgical treatment for macular edema) in the study eye. Systemic steroid treatment was prohibited.

### Outcome assessments and endpoints

Efficacy outcome measures included BCVA by the ETDRS method at each study visit and time-domain OCT (Stratus OCT2 or OCT3) every 3 months. OCT images were evaluated at a central reading center (University of Wisconsin Fundus Photograph Reading Center, Madison, WI, USA) by certified operators masked to study treatment. Safety outcome measures included adverse events and IOP.

The primary efficacy endpoint was the percentage of patients achieving ≥15-letter gain in BCVA from baseline in the study eye at the end of the study. Other predefined efficacy outcomes in the study eye included average change in BCVA from baseline during the study determined with an area-under-the-curve (AUC) approach, time to ≥15-letter gain in BCVA from baseline, and average change in CRT from baseline during the study by OCT (AUC approach). The AUC approach to analysis of BCVA and CRT data takes into account all measurements during the study period, and the average change in BCVA during the study, evaluated with the AUC approach, was used as the primary endpoint of the study for European regulatory authorities.

### Data analysis

All subgroup analyses used data for patients in the DEX implant 0.7 mg (marketed dose) and sham treatment groups. Efficacy subgroup analysis based on any prior treatment, prior laser treatment, and no prior laser treatment was included in the statistical plan for the study. Other subgroup analysis based on prior treatment with intravitreal triamcinolone acetonide, prior treatment with anti-VEGF, and prior treatment with a combination of ≥2 types of therapy among laser, intravitreal triamcinolone acetonide, and anti-VEGF, was post hoc and exploratory.

Efficacy outcomes were evaluated in the intent-to-treat population of all randomized patients. In the analysis of the percentage of patients achieving ≥15-letter gain in BCVA from baseline at study end, missing values were imputed with the last-observation-carried-forward method. The analyses of average change in BCVA and CRT from baseline during the study (AUC approach) and time-to-event data used observed data.

Baseline characteristics of the previously treated patient subgroup were summarized with descriptive statistics. The percentage of patients with ≥15-letter BCVA improvement from baseline was analyzed with the Cochran-Mantel-Haenszel general association test stratified by study, with the 95 % confidence interval of the between-group difference constructed using normal approximation for binary variables. The average change in BCVA or CRT from baseline during the study (AUC approach) was analyzed with analysis of covariance models with treatment and study as fixed effects and baseline BCVA or CRT as the covariate; between-group differences and 95 % confidence intervals were from least-squares means. The Kaplan-Meier method was used to analyze time to ≥15-letter BCVA improvement from baseline, and cumulative response rates were compared using the log-rank test. Safety outcomes were evaluated in the safety population of all patients who received study treatment.

Statistical analysis was performed with SAS version 9.3 (SAS Institute Inc., Cary, NC, USA) using a 2-sided alpha level of 0.05.

## Results

The previously treated subgroup represented 70.4 % (247/351) of patients in the DEX implant 0.7 mg group and 74.6 % (261/350) of patients in the sham group. Baseline demographic and clinical characteristics of previously treated patients were similar for patients in the DEX implant 0.7 mg and sham groups (Table 1). Over 90 % of patients in the previously treated subgroup had received macular laser in the study eye for DME prior to study entry, whereas 23 % had received intravitreal triamcinolone acetonide, and 10 % had received intravitreal anti-VEGF (Table 1). Approximately 23 % of patients in the previously treated subgroup had received at least 2 of these 3 types of therapy for DME in the study eye prior to study entry (Table 1). The median duration of DME in previously treated patients was 20 months in the DEX implant 0.7 mg group and 24 months in the sham group.

**Table 1:** Baseline characteristics of previously treated patients and study eyes (intent-to-treat population)

Characteristic	DEX 0.7	Sham
	*n* = 247	*n* = 261
*Patients*		
Mean age (SD), yr	63.0 (8.3)	63.0 (9.1)
Male, *n* (%)	150 (60.7)	168 (64.4)
Caucasian, *n* (%)	188 (76.1)	192 (73.6)
Mean diabetes duration (SD), yr	16.4 (8.7)	16.2 (9.7)
Type 2 diabetes, *n* (%)	220 (89.1)	238 (91.2)
Mean HbA1c (SD), %	7.5 (1.1)	7.5 (1.0)
≤8 %, *n* (%)	168 (68.0)	189 (72.4)
Mean DME duration (SD), mo	27.3 (26.3)	31.9 (28.6)
*Study eyes*		
Lens status, *n* (%)		
Phakic	182 (73.7)	179 (68.6)
Pseudophakic	65 (26.3)	82 (31.4)
Mean BCVA (SD), ETDRS letters	55.2 (9.6)	56.1 (9.1)
Mean CRT (SD), μm	478 (153)	472 (131)
Prior DME treatment, *n* (%)	247 (100)	261 (100)
Laser	231 (93.5)	243 (93.1)
Intravitreal triamcinolone acetonide	58 (23.5)	61 (23.4)
Intravitreal anti-VEGF	25 (10.1)	26 (10.0)
At least 2 of the 3 types of treatment	61 (24.7)	57 (21.8)
No prior DME treatment, *n* (%)	0 (0)	0 (0)

*BCVA* best-corrected visual acuity, *CRT* central retinal thickness, *DEX 0.7* dexamethasone intravitreal implant 0.7 mg, *DME* diabetic macular edema, *ETDRS* Early Treatment Diabetic Retinopathy Study, *HbA1c* glycosylated hemoglobin, *SD* standard deviation, *VEGF* vascular endothelial growth factor

Three-year study completion rates in the previously treated subgroup were 67.6 % (167/247) for patients in the DEX implant 0.7 mg group and 43.7 % (114/261) for patients in the sham group, similar to those in the overall study population (64.1 % and 43.4 %, respectively). Within the previously treated subgroup, lack of efficacy led to discontinuation of 5.7 % of patients treated with DEX implant 0.7 mg and 24.5 % of patients treated with sham, while adverse events led to discontinuation of 12.1 % of patients treated with DEX implant 0.7 mg and 11.1 % of patients treated with sham. Only 2.8 % and 5.0 % of previously treated patients in the DEX implant 0.7 mg and sham groups, respectively, were lost to follow-up. The mean (standard deviation) number of treatments received over 3 years was 4.1 (1.9) in previously treated patients in the DEX implant 0.7 mg group and 3.2 (2.2) in previously treated patients in the sham group.

Efficacy outcomes were consistently significantly better with DEX implant 0.7 mg than sham in the previously treated subgroup (Table 2). The percentage of previously treated patients achieving ≥15-letter gain in BCVA from baseline at the year 3 or final study visit (primary efficacy endpoint) was 21.5 % in the DEX implant 0.7 mg group versus 11.1 % in the sham group (*P* = 0.002), though the results in the DEX implant 0.7 mg group may have varied based on the type of prior treatment received. The percentage of patients achieving ≥15-letter gain was 27.6 % for patients previously treated with steroids, 28.0 % for patients previously treated with anti-VEGF, and 21.2 % for patients previously treated with laser (Table 3). Previously treated patients in the DEX implant 0.7 mg group also demonstrated greater average improvement in BCVA and CRT from baseline during the study (AUC approach) compared with previously treated patients in the sham group. Mean average improvement in BCVA was +3.2 letters in the DEX implant 0.7 mg group versus +1.5 letters in the sham group (*P* = 0.024), and mean average improvement in CRT was −126 μm in the DEX implant 0.7 mg group versus −39 μm in the sham group (*P* < 0.001). Efficacy outcomes were better with DEX implant 0.7 mg than sham in patient subgroups defined by previous treatment with laser, intravitreal triamcinolone acetonide, or anti-VEGF (Table 3), or by a combination of at least 2 of these 3 types of therapy (Table 4).

**Table 2:** Key efficacy endpoints in previously treated patients

Endpoint	DEX 0.7	Sham	Mean Difference	*P* Value
	*n* = 247	*n* = 261		
Patients with BCVA ≥15-letter improvement from baseline at study end, %	21.5	11.1	10.3	0.002^b^
Mean BCVA average change from baseline during the study (SD), letters^a^	+3.2 (8.7)	+1.5 (7.5)	1.6	0.024^c^
Mean CRT average change from baseline during the study (SD), μm^a^	−126 (131)	−39 (121)	−85	<0.001^c^

^a^Area-under-the-curve approach

^b^Based on the Cochran-Mantel-Haenszel general association test stratified by study

^c^Based on an analysis of covariance model with treatment and study as factors and the baseline value as a covariate

*BCVA* best-corrected visual acuity, *CRT* central retinal thickness, *DEX 0.7* dexamethasone intravitreal implant 0.7 mg; *SD* standard deviation

**Table 3:** Efficacy in Subgroups Defined by Type of Previous Treatment Received

	Prior Steroid		Prior Anti-VEGF		Prior Laser	
Endpoint	DEX 0.7	Sham	DEX 0.7	Sham	DEX 0.7	Sham
	*n* = 58	*n* = 61	*n* = 25	*n* = 26	*n* = 231	*n* = 243
Patients with BCVA ≥15-letter improvement from baseline at study end, %	27.6	8.2	28.0	7.7	21.2	11.9
Mean BCVA average change from baseline during the study (SD), letters^a^	+4.9 (7.4)	−0.6 (8.6)	+4.2 (8.8)	+1.6 (7.6)	+3.1 (8.7)	+1.6 (7.5)
Mean CRT average change from baseline during the study (SD), μm^a^	−121 (150)	−30 (135)	−130 (95)	−42 (123)	−123 (130)	−39 (121)

^a^Area-under-the-curve approach

*BCVA* best-corrected visual acuity, *CRT* central retinal thickness, *DEX 0.7* dexamethasone intravitreal implant 0.7 mg, *SD* standard deviation

**Table 4:** Efficacy in Patients With at Least 2 Types of Previous Treatment^a^

Endpoint	DEX 0.7	Sham	Mean Difference
	*n* = 61	*n* = 57	(95 % CI)
Patients with BCVA ≥15-letter improvement from baseline at study end, %	26.2	8.8	17.5 (4.2, 30.7)^c^
Mean BCVA average change during the study, letters^b^	4.0	−0.3	3.8 (1.1, 6.5)^d^
Mean CRT average change during the study, μm^b^	−108	−26	−111 (−154, −69)^d^

^a^Types of previous treatment were macular laser, intravitreal triamcinolone acetonide, and intravitreal anti-VEGF

^b^Area-under-the-curve approach

^c^Based on the Cochran-Mantel-Haenszel general association test stratified by study

^d^Based on an analysis of covariance model with treatment and study as factors and the baseline value as a covariate

*BCVA* best-corrected visual acuity, *CI* confidence interval, *CRT* central retinal thickness, *DEX 0.7* dexamethasone intravitreal implant 0.7 mg, *VEGF* vascular endothelial growth factor

**Fig. 1:**
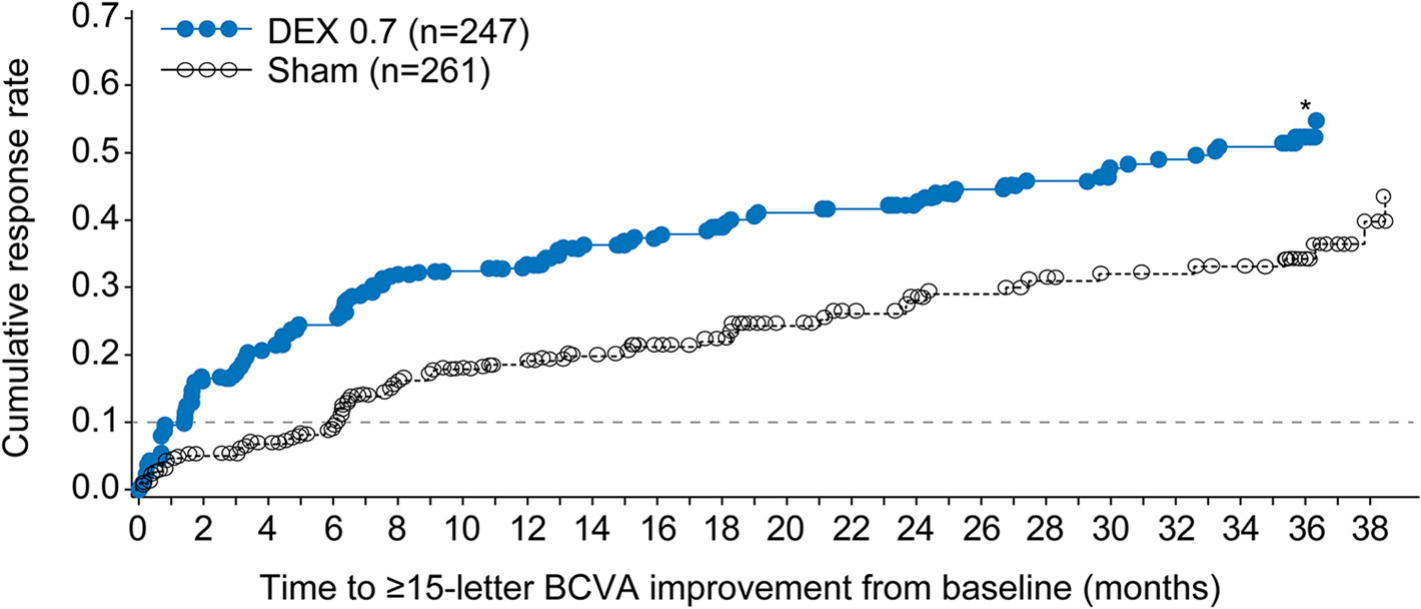
Survival analysis of the time to ≥15-letter improvement in best-corrected visual acuity (BCVA) from baseline. *P < 0.001 vs sham (log-rank test comparing cumulative response rate curves over time). DEX 0.7 = dexamethasone intravitreal implant 0.7 mg

Within the subgroup of patients with any previous treatment for DME, patients in the DEX implant 0.7 mg group showed significantly earlier ≥15-letter gain in BCVA from baseline compared with patients in the sham group (*P* < 0.001, Fig. 1). Separation of the cumulative response rate curves was evident before the first efficacy visit. The time to the 10th percentile cumulative response was 41 days with DEX 0.7 mg versus 184 days with sham.

**Fig. 2:**
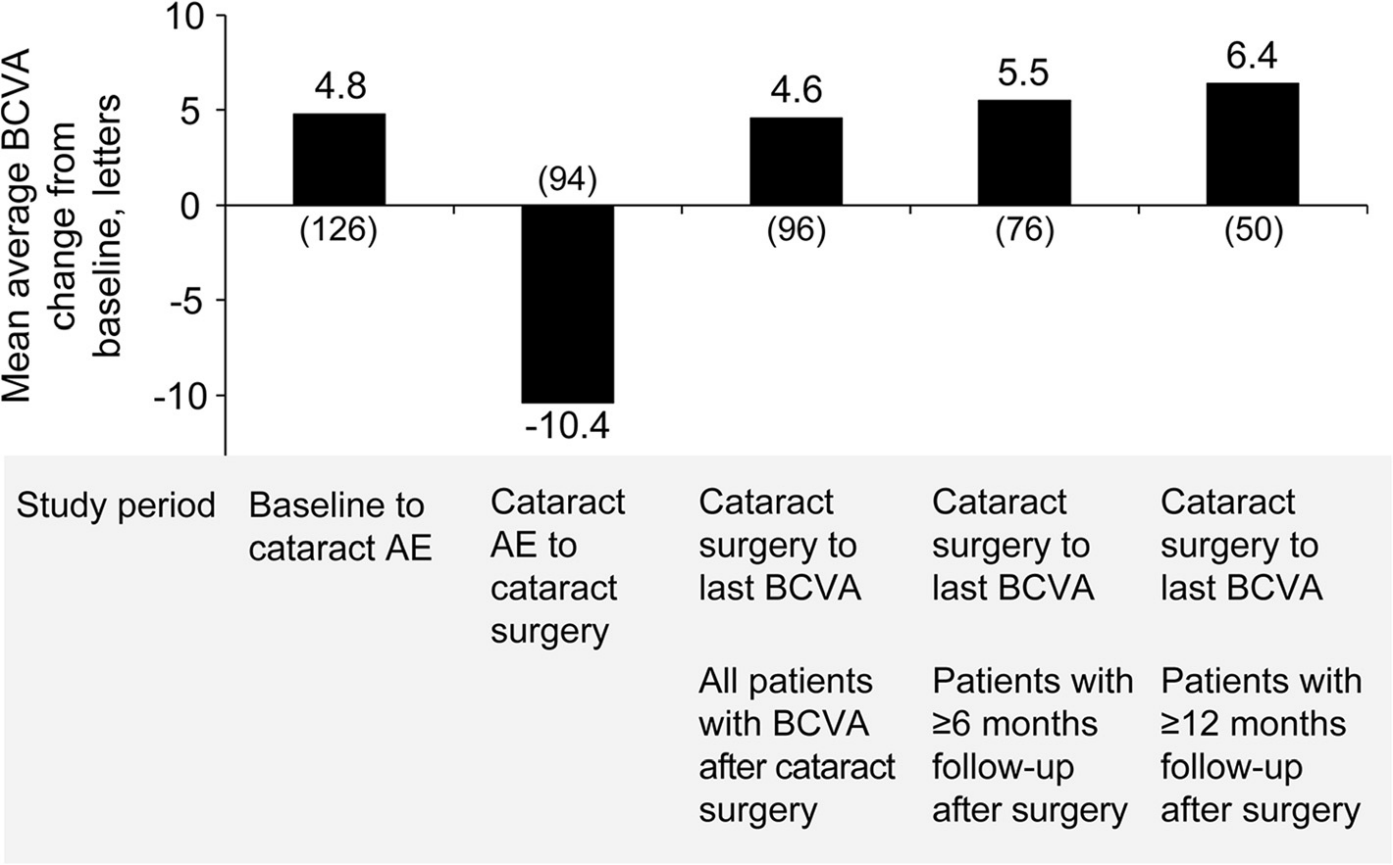
Mean average best-corrected visual acuity (BCVA) change from baseline before and after cataract surgery. Results are shown for previously treated patients with cataract-related adverse events (AEs) in the dexamethasone intravitreal implant 0.7 mg group. Numbers in parentheses indicate number of patients

The safety profile of DEX implant 0.7 mg in previously treated patients was similar to its safety profile in the total study population [21]. The most common adverse events in the previously treated subgroup were steroid-related increases in IOP and cataract (Table 5). Within the previously treated subgroup, cataract-related adverse events were reported in 70.3 % of baseline phakic patients in the DEX implant 0.7 mg group. In patients who had cataract-related adverse events, vision gains were restored following cataract surgery (Fig. 2).

**Table 5:** Incidence of Adverse Events (Safety Population)

	Previously Treated Patients		Total Study Population	
Incidence, %	DEX 0.7	Sham	DEX 0.7	Sham
	*n* = 247	*n* = 261	*n* = 347	*n* = 350
Serious ocular AE	6.9	0.8	6.9	1.1
IOP-related AE^a^	38.1	4.6	36.0	5.1
Cataract-related AE (incidence in phakic eyes)	70.3	20.1	67.9	20.4

^a^Any adverse event (AE) related to increased intraocular pressure or glaucoma

*AE* adverse event, *DEX 0.7* dexamethasone intravitreal implant 0.7 mg, *IOP* intraocular pressure

## Discussion

Preplanned subgroup analysis of the MEAD study results showed that DEX implant 0.7 mg significantly improved visual and anatomic outcomes in patients with a history of previous medical or laser treatment for DME. Exploratory analysis of outcomes in patient subgroups defined by previous treatment of DME with intraocular triamcinolone acetonide, anti-VEGF, or at least 2 types of therapy (among laser, intraocular steroid, and anti-VEGF) also showed benefit of DEX implant 0.7 mg treatment relative to sham. Safety findings for DEX implant in the previously treated subgroup were similar to those in the total patient population.

Most of the patients enrolled in the MEAD study had persistent edema and vision loss despite prior therapy. Because the study was sham controlled, investigators were unlikely to allow patients who were adequately responsive to available treatments to enter the study. Therefore, the previously treated subgroup represented a difficult-to-treat population. Among the previously treated patients in the DEX implant 0.7 mg and sham groups, the mean duration of edema at study entry was approximately 2.5 years, and over 90 % had been treated previously with laser for DME in the study eye. Results of the subgroup analysis demonstrated the efficacy of DEX implant in this difficult-to-treat population. Efficacy outcomes in the previously treated subgroup of patients were very similar to those in the total study population [21]. Within the previously treated subgroup, the percentage of patients with ≥15-letter BCVA gain at the end of the study was significantly higher with DEX implant 0.7 mg than with sham, and the average change in BCVA and CRT from baseline during the study (AUC approach) was significantly greater with DEX implant 0.7 mg than with sham.

In the total study population, analysis of BCVA changes from baseline at each visit showed diminished treatment effect by 6 months postinjection [21]. Furthermore, in a recent prospective study evaluating visual and anatomic outcomes after DEX implant treatment in retinal diseases including DME, the optimal interval for DEX implant retreatment, based on efficacy and safety outcomes, was determined to be 20 weeks (4 to 5 months) [23]. Thus, it is likely that more frequent dosing of DEX implant than the ≥6-month intervals used in the MEAD study would have provided more consistent improvement in BCVA over time and at study end, and the MEAD study outcomes may underestimate the true value of DEX implant in DME.

Efficacy of DEX implant in previously treated patients in the MEAD study, reported here, is consistent with the results of previous small studies of DEX implant treatment in patients with persistent DME despite prior therapy [24, 25]. In those studies, DEX implant improved BCVA and foveal thickness in patients with edema that was refractory to laser, intraocular steroid, or anti-VEGF treatment [24, 25]. Although anti-VEGF is often used as first-line treatment of DME, not all patients respond to anti-VEGF. The number of patients in the MEAD study who had been previously treated with anti-VEGF was small, but the subgroup analysis results nonetheless suggest that DEX implant effectively improved BCVA and CRT in patients previously treated with anti-VEGF. These results are consistent with the previous report of DEX implant effectiveness in the treatment of DME that had not responded to 3 monthly injections of intravitreal bevacizumab anti-VEGF therapy [25]. The multifactorial nature of DME [2] may explain why some patients are refractive to anti-VEGF treatment but respond to steroids.

The most common adverse event associated with DEX implant treatment in the MEAD study was cataract [21]. Although phakic eyes are at high risk of cataract progression after multiple DEX implant injections, treatment with DEX implant may be justifiable in phakic patients who have not responded to other treatment, because persistent DME can lead to irreversible vision loss, whereas patients who develop cataract during DEX implant treatment recover vision gain after cataract extraction. The analysis of BCVA gain in patients after cataract surgery showed a trend for greater improvement in BCVA with longer follow-up. These results suggest that the recovery time from cataract surgery for patients who underwent cataract extraction near (e.g., within 3 months of) the end of the study may not have been long enough for them to regain treatment benefit by the study end. Timely extraction of cataracts is recommended during DEX implant treatment to maximize visual gains.

There are several limitations of the present analysis. Although patients could be categorized by their previous treatment for DME, information regarding the number of prior laser, anti-VEGF, and steroid treatments received by each patient was not collected. Because the study began in 2004, relatively few patients had received previous treatment with anti-VEGF agents. Also, patients with prior pars plana vitrectomy, a surgical option for DME in some patients, were excluded. However, it has been reported that DEX implant is also effective in the treatment of DME in eyes with prior vitrectomy [26, 27]. The study completion rates were relatively low, especially in the sham group, because of the MEAD study design requirement for patients to exit the study before receiving any escape therapy [21]. This study requirement prevented any confounding effects of concomitant therapy from influencing the study results, but also led to an increased rate of patient discontinuations, which can bias study results if the remaining patients are not representative of the larger randomized patient population. Among previously treated patients who received DEX implant 0.7 mg, the discontinuation rate was approximately 32 %, compared with discontinuation rates of approximately 20 %–30 % reported in 3-year studies of medical therapy in DME that allowed patients to receive rescue therapy and continue in the study [28, 29]. Patients treated with sham procedure discontinued earlier and at a higher rate than patients treated with DEX implant because of lack of efficacy and need for escape therapy. Appropriate statistical methods were used to deal with patient dropouts and missing values in the data analysis.

## Conclusion

This subgroup analysis has shown beneficial effects of DEX implant 0.7 mg treatment on visual and anatomic outcomes in patients with previously treated DME. Significant improvement was seen with a mean of 4.1 DEX implant injections over 3 years. The data suggest that DEX implant may have a role in the treatment of DME in the substantial number of patients who fail to respond to standard therapy. A randomized clinical trial comparing DEX implant and continued anti-VEGF therapy is warranted to more definitively evaluate the benefit of DEX implant treatment in anti-VEGF nonresponders.
